# Electronic differentiation competes with transition state sensitivity in palladium-catalyzed allylic substitutions

**DOI:** 10.1186/1860-5397-3-36

**Published:** 2007-10-26

**Authors:** Dominik A Lange, Bernd Goldfuss

**Affiliations:** 1 Institut für Organische Chemie, Universität zu Köln, D-50939 Köln, Germany

## Abstract

Electronic differentiations in Pd-catalyzed allylic substitutions are assessed computationally from transition structure models with electronically modified phospha-benzene-pyridine ligands. Although donor/acceptor substitutions at P and N ligand sites were expected to increase the site selectivity, i.e. the preference for "*trans* to P" attack at the allylic intermediate, acceptor/acceptor substitution yields the highest selectivity. Energetic and geometrical analyses of transition structures show that the *sensitivity* for electronic differentiation is crucial for this site selectivity. Early transition structures with acceptor substituted ligands give rise to more intensive Pd-allyl interactions, which transfer electronic P,N differentiation of the ligand more efficiently to the allyl termini and hence yield higher site selectivities.

## Introduction

**Scheme 1:**
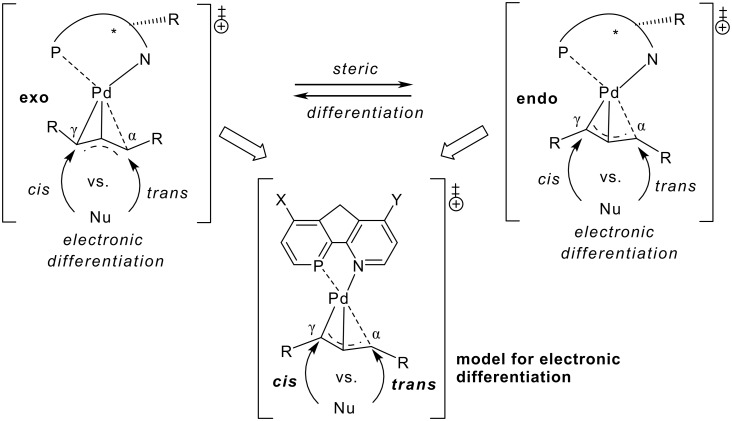
Electronic and steric differentiations provide the basis for the high selectivity of P,N-ligands in Pd-catalyzed allylic substitutions. Effects are studied with P-N-model ligands with *para*-substituted, coplanar phosphabenzene and pyridine moieties.

Palladium-catalyzed allylic substitutions allow very selective and mild allylations of C-,N- and O-nucleophiles. [1–13] The selectivity derives from steric and electronic properties of substrate and catalyst structures. "Side arm guidance" of nucleophiles with multifunctional phosphinoferrocenes [14–18] or "chiral pockets" in C_2_-symmetric diphosphanes based on 2-(diphenyl-phosphino)benzoic acid amides [19–22] were applied especially successfully. Chiral P,N-ligands (e.g. phosphinooxazolines, phox) [23–27] provide in addition to steric control the possibility for "electronic differentiation", originating from the *trans*-influence [28] of different donor atoms. Nucleophiles (e.g. dimethylmalonate) normally favour addition to the "*trans* to phosphorus" position at the Pd-η^3^-allylic intermediate (Scheme 1). [29–42] This "*trans* to P" rule is supported by X-ray and computational analyses of Pd-η^3^-allylic intermediates, which exhibit longer and hence weaker Pd-C_allyl_ bonds *trans* to P (i.e. the stronger π-acceptor vs. N) and hence are more susceptible to nucleophilic attack (Scheme 1). [29–41] This electronic differentiation contributes to the high selectivity in Pd-catalyzed asymmetric allylic substitutions[19] and provides also an explanation for α-memory effects. [42–43] Computational model systems for P,N-ligands, i.e. PH_3_ and *para*-substituted pyridines, have shown that *cis-trans* differentiations, i.e. the electronic site selectivity, of nucleophilic additions to Pd-η^3^-allylic intermediates is highest for electron poor pyridine ligands.[45]

To further explore origins of site selectivities based on electronic differentiations in Pd-catalyzed allylic substitutions, we here employ a more advanced model system with phosphabenzene, [45–48] and pyridine moieties for the crucial step of Pd-catalyzed allylic substitutions. Both P- and N-coordination sites are tuned electronically with *para*-substituents to reveal energetic and geometrical effects on cis- vs. trans- additions of nucleophiles to the Pd-η^3^-allylic intermediates (Scheme 1).

## Results and Discussion

**Scheme 2:**
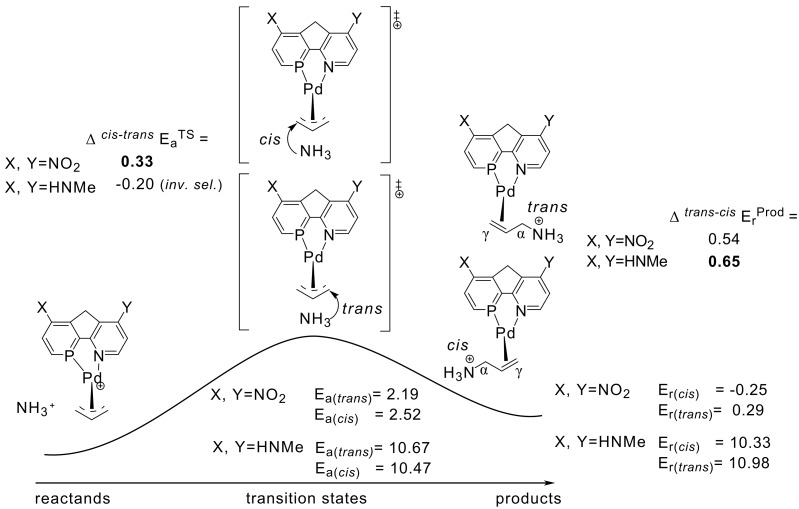
Activation (ΔE_a_) and reaction (ΔE_r_) energies (kcal mol^-1^), computed for the P,N-ligand model with tuneable electronic differentiation.

Electron donating or withdrawing groups (i. e. X, Y = HNMe, H, NO_2_) in *para*-positions of phosphabenzene (X) and pyridine (Y) units tune electronic characteristics of P,N-ligand models in Pd-catalyzed allylic substitutions (Scheme 1). The phosphabenzene and pyridine moieties are linked via C_ar_-C_ar_ bonds and a methylene bridge retains planarity and limits conformational flexibility. NHMe rather than higher substituted NMe_2_ was employed as donor group, to retain lp-aryl conjugation. Ammonia serves as model nucleophile and attacks the Pd-η^3^-allylic intermediate *cis* or *trans* to phosphorus. This *cis* vs. *trans* site selectivity is employed as measure for electronic differentiation induced by the ligand system (Scheme 2).

**Figure 1:**
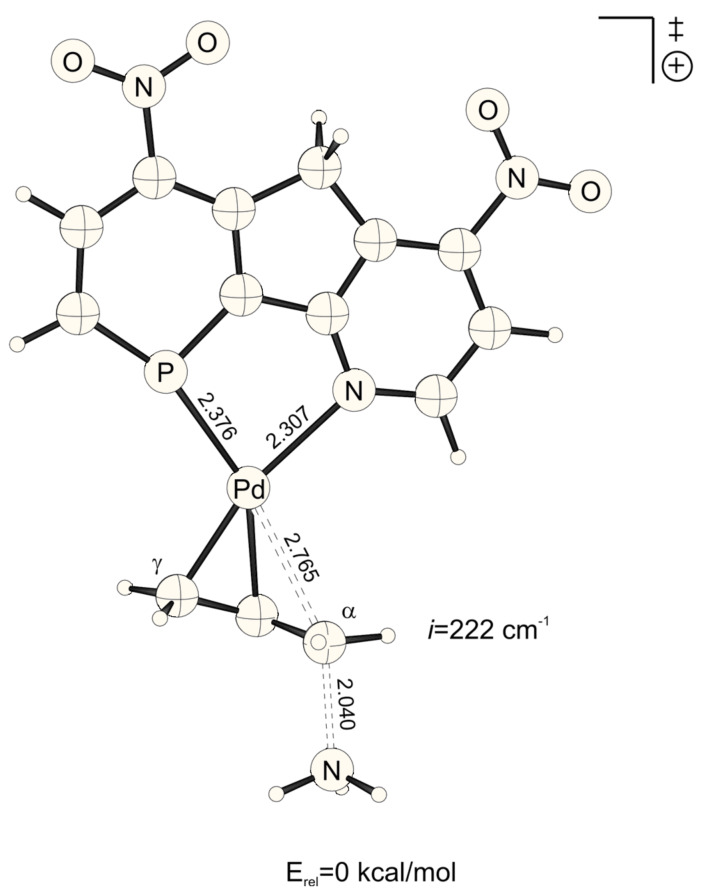
Transition structure for the energetically favored *trans* to phosphorus addition of ammonia at the Pd-η^3^-allylic intermediate (B3LYP/6-31G* (C, H, N, P, O), /SDD (Pd)). Bond distances are given in Å.

**Figure 2:**
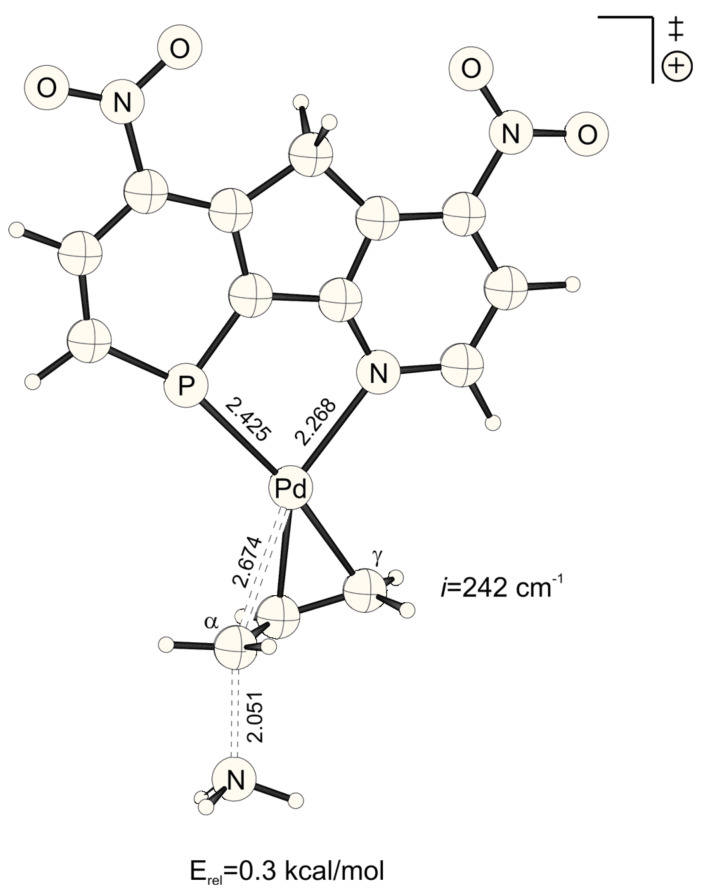
Transition structure for the energetically disfavored *cis* to phosphorus addition of ammonia at the Pd-η^3^-allylic intermediate (B3LYP/6-31G* (C, H, N, P, O), /SDD (Pd)). Bond distances are given in Å.

**Figure 3:**
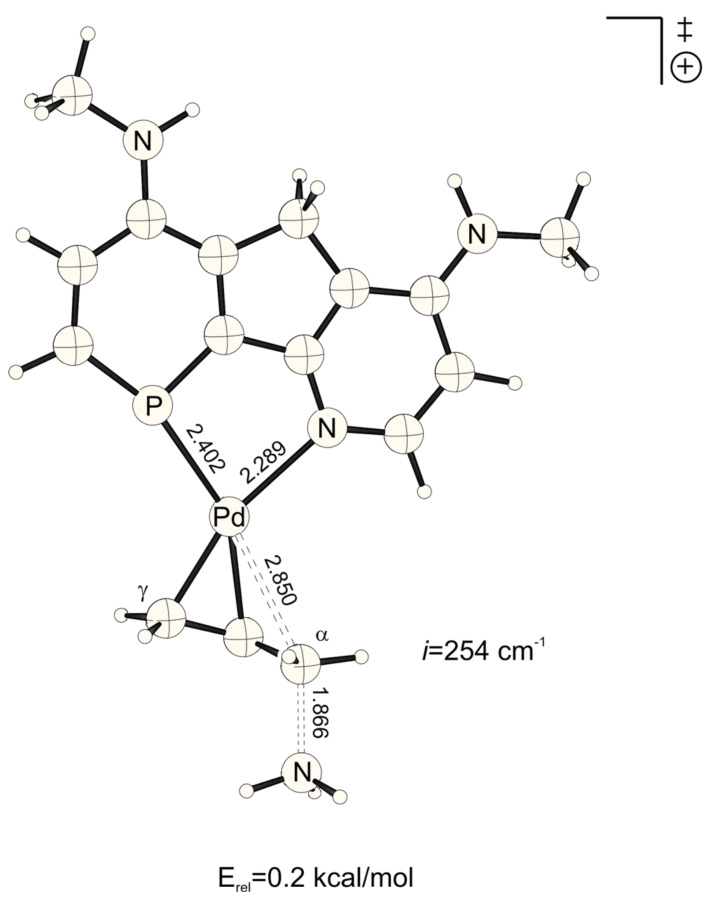
Transition structure for the energetically disfavored *trans* to phosphorus addition of ammonia at the Pd-η^3^-allylic intermediate (B3LYP/6-31G* (C, H, N, P, O), /SDD (Pd)). Bond distances are given in Å.

**Figure 4:**
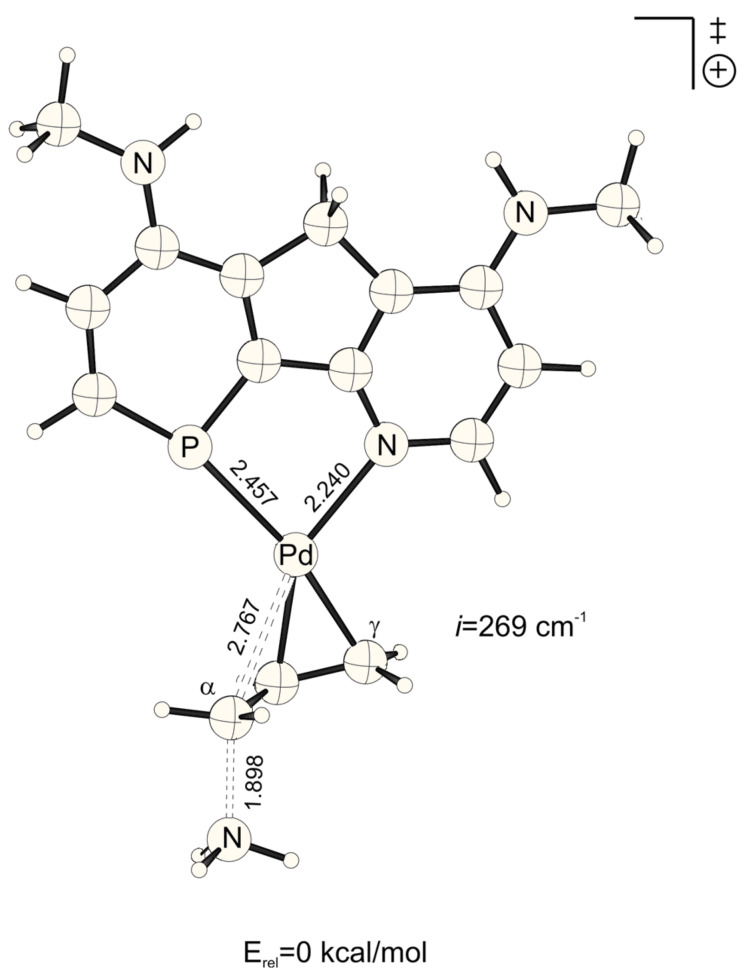
Transition structure for the energetically favored *cis* to phosphorus addition of ammonia at the Pd-η^3^-allylic intermediate (B3LYP/6-31G* (C, H, N, P, O), /SDD (Pd)). Bond distances are given in Å.

The lowest activation energies (E_a_, Table 1) for ammonia addition to the Pd-η3-allylic intermediate are apparent for strong electron withdrawing *para*-substituted phosphabenzene and pyridine units, i.e. X, Y = NO_2_ (Figure 1 and Figure 2, E_a_*^trans^* = 2.19, E_a_*^cis^* = 2.52 kcal mol^-1^, Table 1). The highest activation energies result from electron donating amino groups X, Y = NHMe (Figure 3 and Figure 4, E_a_*^trans^* = 10.67, E_a_*^cis^* = 10.47 kcal mol^-1^, Table 1, Scheme 2). Such electronic tunings of the ligands strongly affect the reactivity and give rise to increased or decreased electrophilicity of Pd-allyl intermediates.

**Table 1:** Activation (E_a_) and reaction energies (E_r_) reflecting electronic differentiations in transition structures (ΔE_a_*^cis-trans^*) and Pd-ene products relative to Pd-allyl and NH_3_ reactands (pb = phosphabenzene; py = pyridine moieties)^[a]^

pb-X	py-Y	E_a_	TS	ΔE_a_^TS^	E_r_^Prod^	ΔE_r_^Prod^

H	HNMe	*cis*	8.55	0.03	7.81	0.55
		*trans*	8.52		8.36	
H	H	*cis*	6.38	0.17	5.14	0.52
		*trans*	6.21		5.67	
H	NO_2_	*cis*	4.47	0.27	2.48	0.54
		*trans*	4.20		3.02	

HNMe	HNMe	*cis*	10.47	***-0.20*** ^[b]^	10.33	**0.65**
		*trans*	10.67		10.98	
HNMe	H	*cis*	8.43	-0.03^[b]^	7.80	0.60
		*trans*	8.46		8.40	
HNMe	NO_2_	*cis*	6.61	0.10	5.34	**0.65**
		*trans*	6.51		5.99	

NO_2_	HNMe	*cis*	6.34	0.08	5.05	0.53
		*trans*	6.26		5.58	
NO_2_	H	*cis*	4.24	0.23	2.26	0.43
		*trans*	4.01		2.70	
NO_2_	NO_2_	*cis*	2.52	***0.33***	-0.25^[c]^	0.54
		*trans*	2.19		0.29	

[a] B3LYP/6-31G* (C, H, N, P, O), /SDD (Pd) optimized structures. Energies include ZPE corrections scaled by 0.9806; [b] Negative ΔE_a_^TS^ with E_a_^cis^ < E_a_*^trans^*; [c] exothermic reaction energy.

The reaction energies (E_r_) for ammonia addition to the Pd-η3-allylic intermediate show a similar preference: Pd-ene-adduct formation is favoured most for X, Y = NO_2_ (E_r_*^trans^* = 0.29, E_r_*^cis^* = -0.25 kcal mol^-1^) and becomes most unfavourable (i.e. endothermic) for X, Y = NHMe (E_r_*^trans^* = 10.98, E_r_*^cis^* = 10.33 kcal mol^-1^, Table 1, Scheme 2). This points to a more π-donating character of the ene product relative to the allyl-cation reactant.

**Figure 5:**
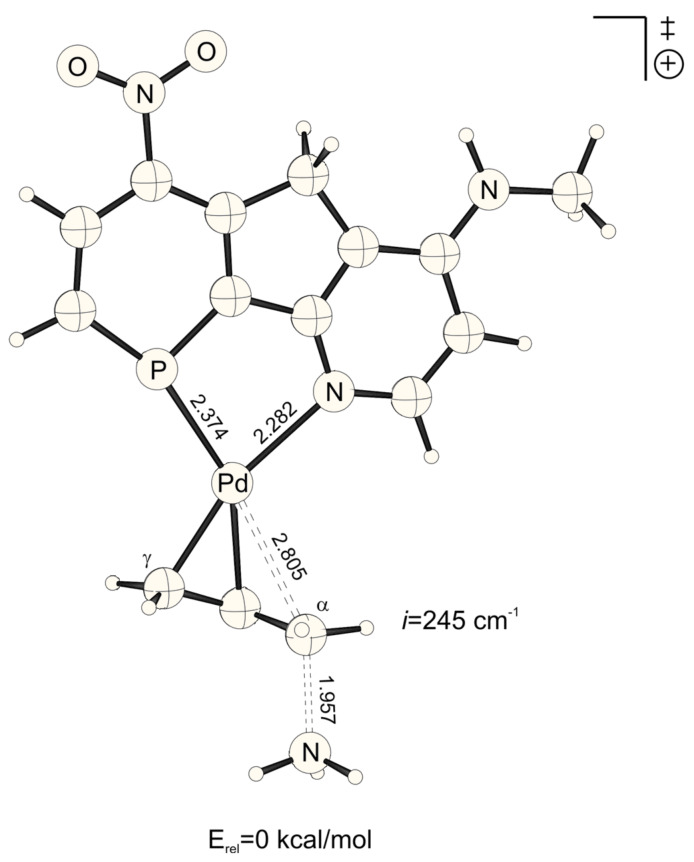
Transition structure for the energetically favored *trans* to phosphorus addition of ammonia at the Pd-η^3^-allylic intermediate (B3LYP/6-31G* (C, H, N, P, O), /SDD (Pd)). Bond distances are given in Å.

**Figure 6:**
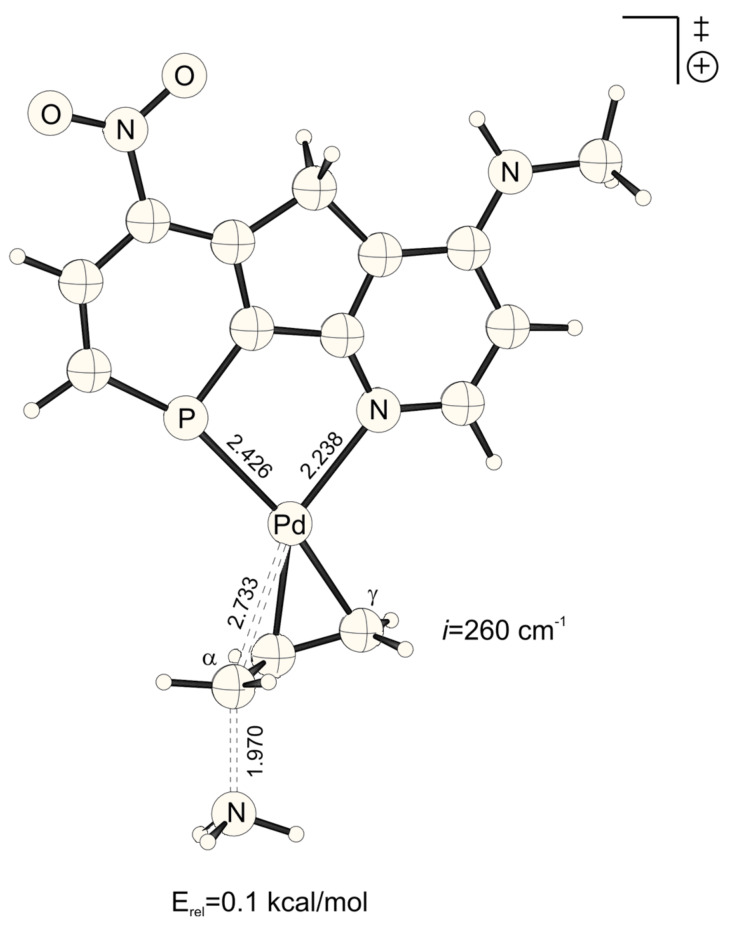
Transition structure for the energetically disfavored *cis* to phosphorus addition of ammonia at the Pd-η^3^-allylic intermediate (B3LYP/6-31G* (C, H, N, P, O), /SDD (Pd)). Bond distances are given in Å.

**Figure 7:**
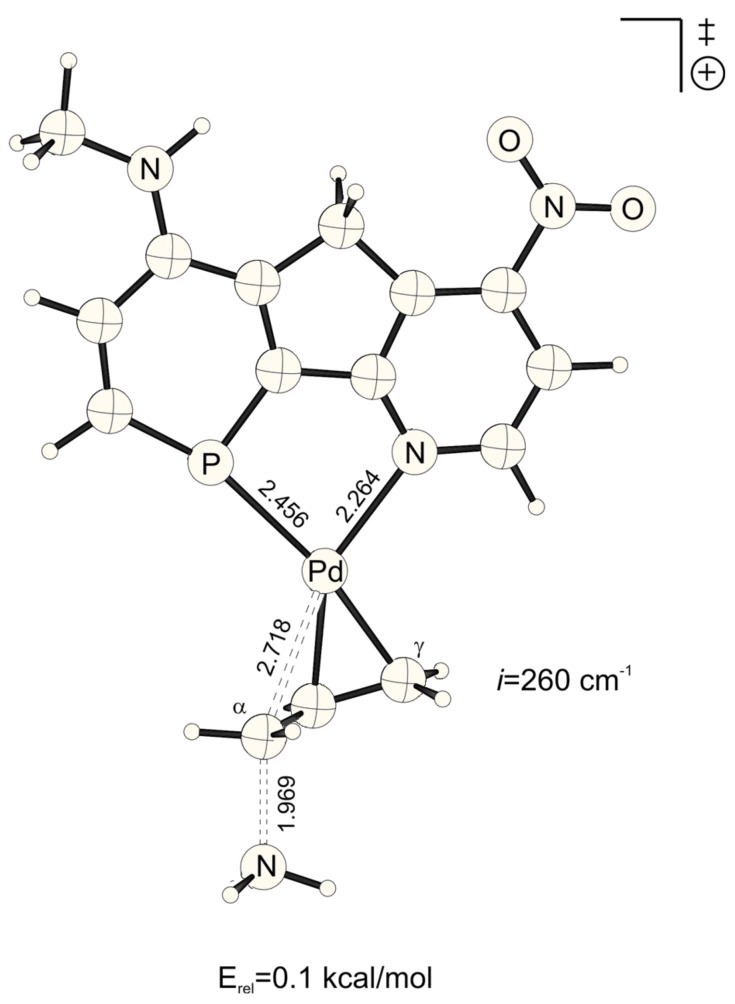
Transition structure for the energetically disfavored *cis* to phosphorus addition of ammonia at the Pd-η^3^-allylic intermediate (B3LYP/6-31G* (C, H, N, P, O), /SDD (Pd)). Bond distances are given in Å.

**Figure 8:**
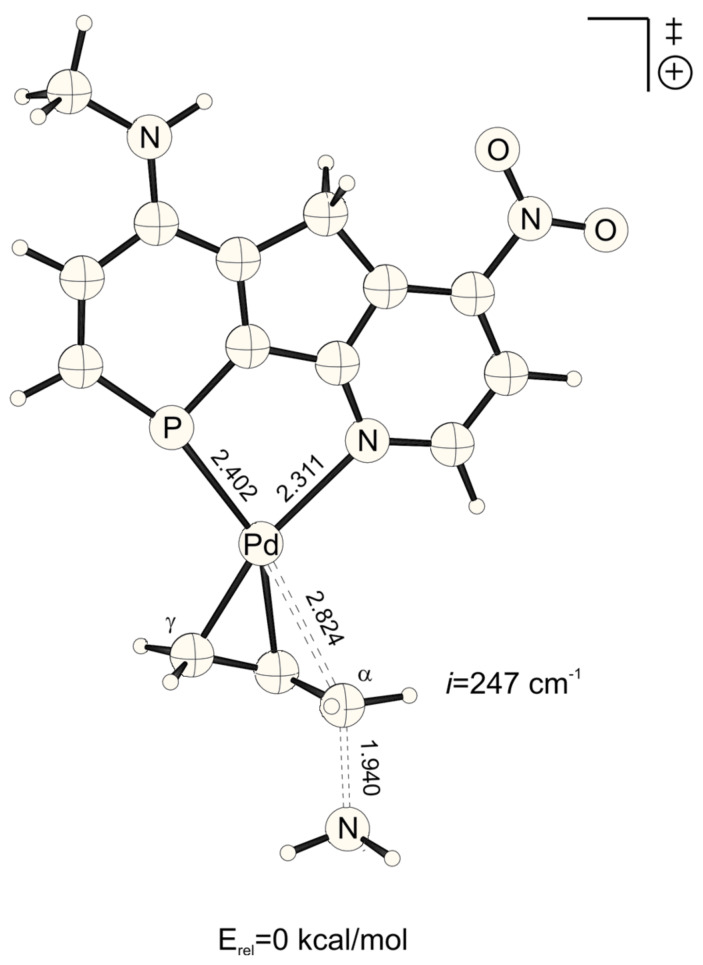
Transition structure for the energetically favored trans to phosphorus addition of ammonia at the Pd-η^3^-allylic intermediate (B3LYP/6-31G* (C, H, N, P, O), /SDD (Pd)). Bond distances are given in Å.

In agreement with the "*trans* to phosphorus" rule, [23–28] attack of ammonia is preferred for most X, Y combinations *trans* to P, due to the stronger π*/σ* acidity at P in phosphabenzene relative to N in pyridine (Table 1).[44] Surprisingly however, this electronic site selectivity, as it is measured from relative energies of the transition structures (ΔE_a_^TS^), is not largest for different X, Y donor-acceptor combinations (Figure 5, Figure 6, Figure 7 and Figure 8), but is highest for X and Y = NO_2_ (ΔE_a_^TS^ = 0.33 kcal mol^-1^, Table 1). Likewise, the smallest electronic site "*trans* to P" selectivity is not found for X, Y donor-acceptor combinations, but for strong donating X and Y = NHMe. Here, the selectivity is so low, that it even inverts to "*cis* to P" (ΔE_a_^TS^ = -0.20 kcal mol^-1^, Table 1).

**Figure 9:**
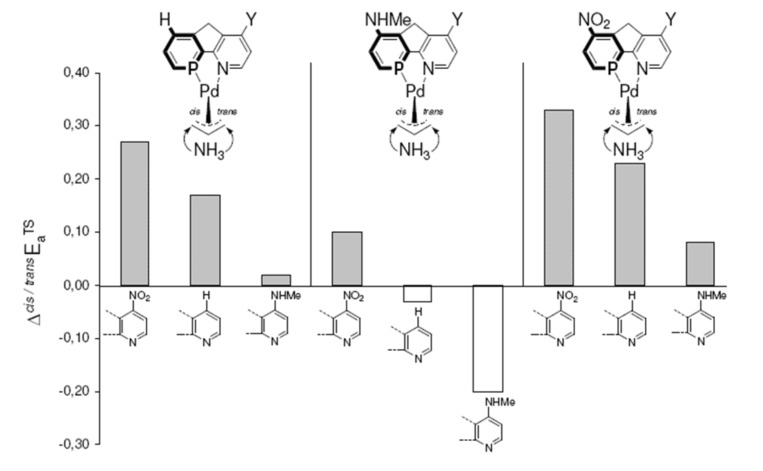
For each phosphabenzene moiety, the site selectivities ΔE_a_^TS^ increase with more electron withdrawing pyridine substituents (Y) in the order HNMe < H < NO_2_ (cf. Table 1).

For each phosphabenzene moiety with X = H or NHMe or NO_2_, the "*trans* to P" site selectivity ΔE_a_^TS^ increases for pyridine substituents Y in the order NHMe < H < NO_2_ (Figure 9, Table 1). Hence, there is apparently an additional effect, which controls the site selectivity ΔE_a_^TS^ besides the electronic donor vs. acceptor properties of different ligand atoms, i.e. P vs. N. Via this effect; electron withdrawing groups (e.g. NO_2_) give rise to the highest site-selectivities.

NO_2_-substituted ligands give rise to earlier transition structures with longer (forming) H_3_N-C_α_ bonds (Table 2, Figure 1 to Figure 8), e.g. *trans*-TS with X = Y = NO_2_: H_3_N-C_α_ = 2.04 Å (Figure 1). In contrast, amino-donor substitution leads to later transition structures with shorter H_3_N-C_α_ distances, e.g. *trans*-TS with X = Y = NHMe: H_3_N-C_α_ = 1.866 Å (Figure 3). This agrees with the more electrophilic properties of cationic Pd-allyl intermediates induced by electron withdrawing ligands.

**Table 2:** H_3_N-C_α_, H_3_N^+^-C_α_ and Pd-C_α_ distances (Å) of transition states and Pd-ene product complexes (pb = phosphabenzene; py = pyridine)^[a]^

		Transition structures			Pd-ene product complexes

Pb-X	py-Y	Pd-C_α_		H_3_N-C_α_	H_3_N^+^-C_α_

H	HNMe	*cis*	2.754	1.930	1.594
		*trans*	2.834	1.906	1.604
H	H	*cis*	2.728	1.968	1.588
		*trans*	2.815	1.947	1.598
H	NO_2_	*cis*	2.696	2.010	1.583
		*trans*	2.797	1.989	1.592

HNMe	HNMe	*cis*	2.767	1.898	1.598
		*trans*	2.850	1.866	1.611
HNMe	H	*cis*	2.745	1.932	1.593
		*trans*	2.840	1.902	1.603
HNMe	NO_2_	*cis*	2.718	1.969	1.588
		*trans*	2.824	1.940	1.598

NO_2_	HNMe	*cis*	2.733	1.970	1.587
		*trans*	2.805	1.957	1.596
NO_2_	H	*cis*	2.703	2.012	1.582
		*trans*	2.787	1.997	1.590
NO_2_	NO_2_	*cis*	2.674	2.051	1.578
		*trans*	2.765	2.040	1.586

[a] B3LYP/6-31G* (C, H, N, P, O), /SDD (Pd) optimized structures. Energies include ZPE corrections scaled by 0.9806.

**Figure 10:**
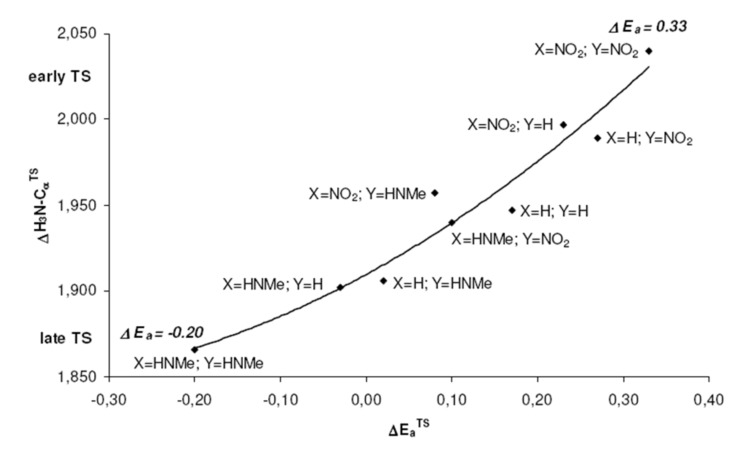
Higher site selectivities, i.e. larger ΔE_a_^TS^ values, are found for earlier transition structures with larger H_3_N-C_α_ distances.

These positions on the reaction coordinate indeed correspond to the site selectivity of the transition structures, i.e. ΔE_a_^TS^: earlier transition structures have higher, later transition structures exhibit lower "*trans* to P" selectivities (Figure 10).

**Figure 11:**
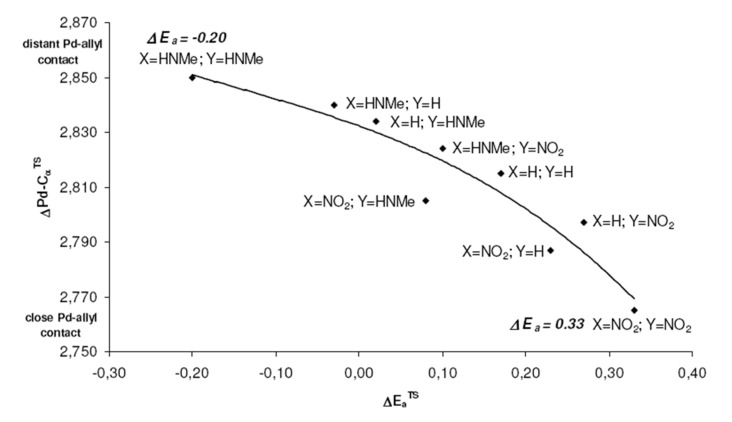
Higher site selectivities, i.e. larger ΔE_a_^TS^ values, are found for transition structures with closer, more intense Pd-C_α_ contacts.

The distance between Pd and the allylic systems decreases from early (allyl cation like) to late (ene like) positions on the reaction coordinate. A closer, more intense Pd-C_α_ contact (e.g. 2.674 Å, Figure 2, Table 2) stronger delivers electronic differentiation of the ligand, and hence "*trans* to P" selectivity. Hence, higher electronic site selectivity closely corresponds to intense Pd-allyl interactions with short Pd-C_α_ distances (Figure 11).

Apparently, the positions on the reaction coordinate influence the site selectivity even stronger than the electronic differentiation between P and N ligand atoms: No substitution (X = Y = H) gives rise to even higher ΔE_a_^TS^ than more pronounced electronic differentiations with X, Y = NO_2_ or NHMe (Figure 11), due to higher TS-sensitivity originating from closer Pd-allyl contact.

## Conclusion

In Pd-catalyzed allylic substitutions, the electronic site selectivity, i.e. the preference for "*trans* to P" addition, is affected by the intrinsic electronic differentiation of the ligand atoms, e.g. P vs. N. However, the sensitivity for this electronic differentiation depends on the intensity of the Pd-allyl interaction. A close Pd-allyl distance in an early, allyl cation like transition structure delivers the electronic differentiation of the ligand system more efficiently to the allylic termini (C_α_) than a more distant Pd-allyl (more ene like) unit of a late transition structure. Electron withdrawing (e.g. NO_2_) substituents in the ligand system generate earlier transition structures with more intense Pd-allyl interactions and higher sensitivity for electronic differentiations. Hence, both intrinsic electronic differentiation in the ligand and high TS-sensitivity appear to be crucial for high site-selectivity in Pd-catalyzed allylic substitutions.

## Computational details

All structures were fully optimized and characterized by frequency computations as minima or transition structures using Gaussian 03[49] with standard basis sets [50–51] and the B3LYP [52–55] hybrid-DFT method. Zero point energies and thermochemical analysis were scaled by 0.9806.[56]
